# Facial Neuralgia—Catalepsy

**Published:** 1831

**Authors:** 


### XXXVII.

### Facial Neuralgia—Catalepsy.

I. *Facial Neuralgia cured by Spontaneous Ptyalism.* By Dr. Carresi. A female, aged 52 years, became affected with severe facial neuralgia after exposure to cold. It continued for four months, and so completely resisted every remedy employed, that she gave up medicine, and trusted to Nature. In about a fortnight after this determination, a spontaneous flow of saliva took place, great in quantity, and sweet as honey in quality. It increased so much in three days that it appeared to flow from the stomach rather than from the salivary glands. During this extraordinary ptyalism, the bowels were constipated, and the urine very scanty, as well as all the other secretions. The facial pain diminished in proportion as the salivation increased:—both ultimately ceased.—*Annali Universale de Medicina.*

II. CATALEPSY ACCIDENTALLY CURED.

In the journal above-mentioned, for October, 1830, there are related two cases of catalepsy, one in a female, the other in a male, which yielded to accidental hæmorrhage—in one case, from a cut on the head—in the other, from epistaxis. The first case was that of a young girl, ten years of age, who became cataleptic, the paroxysms returning at shorter and shorter intervals, and resisting all medicines. In one of these attacks the girl dashed her head against a sharp stone, when a profuse hæmorrhage instantly ensued. This hæmorrhage not only put an end to the attack, but prevented all returns of the disease.

The other case was that of a young farmer, 20 years of age, and of a melancholic temperament, who, after some severe mental distress, became affected with cataleptic attacks, complicated frequently with delirium and somnambulism. Repeated bleedings, leeches, baths, blisters, and various other means were employed in vain. At length a profuse nasal hæmorrhage took place spontaneously, and put an end to the attacks.

---

